# Strategies to Improve the Biosynthesis of β-Lactam Antibiotics by Penicillin G Acylase: Progress and Prospects

**DOI:** 10.3389/fbioe.2022.936487

**Published:** 2022-07-18

**Authors:** Xin Pan, Lei Xu, Yaru Li, Sihua Wu, Yong Wu, Wenping Wei

**Affiliations:** ^1^ Department of Cardiology, Central Laboratory, The Affiliated Hospital of Yangzhou University, Yangzhou University, Yangzhou, China; ^2^ State Key Laboratory of Biochemical Engineering, Institute of Process Engineering, Chinese Academy of Sciences, Beijing, China; ^3^ Division of Molecular Science, Graduate School of Science and Technology, Gunma University, Kiryu, Japan

**Keywords:** β-Lactam antibiotics, biosynthesis, penicillin G acylase, enzymatic property, reaction system

## Abstract

β-Lactam antibiotics are widely used anti-infection drugs that are traditionally synthesized via a chemical process. In recent years, with the growing demand for green alternatives, scientists have turned to enzymatic synthesis. Penicillin G acylase (PGA) is the second most commercially used enzyme worldwide with both hydrolytic and synthetic activities toward antibiotics, which has been used to manufacture the key antibiotic nucleus on an industrial level. However, the large-scale application of PGA-catalyzed antibiotics biosynthesis is still in the experimental stage because of some key limitations, such as low substrate concentration, unsatisfactory yield, and lack of superior biocatalysts. This paper systematically reviews the strategies adopted to improve the biosynthesis of β-lactam antibiotics by adjusting the enzymatic property and manipulating the reaction system in recent 20 years, including mining of enzymes, protein engineering, solvent engineering, *in situ* product removal, and one-pot reaction cascade. These advances will provide important guidelines for the future use of enzymatic synthesis in the industrial production of β-lactam antibiotics.

## Introduction

With annual sales of more than 15 billion dollars, β-lactam antibiotics are important anti-infection drugs that account for about 65% of the global antibiotics market share (Chandel et al., 2008; Rodriguez-Herrera et al., 2019). Penicillins, cephalosporins, and other antibiotics that comprise a β-lactam ring in their chemical structures can inhibit the synthesis of bacterial cell walls by binding to penicillin binding proteins (Alekseev, 2010). Unfortunately, the indiscriminate use of antibiotics enables surviving bacteria to develop resistance to antibacterial drugs (Buchholz, 2016; Chaudhary, 2016). Currently, the majority of existing natural antibiotics are used to produce key intermediates of semisynthetic penicillins and cephalosporins, and only a small fraction of these drugs are directly applied in clinical treatment (Parmar et al., 2000). Compared with natural antibiotics, semisynthetic penicillins and cephalosporins have greater advantages, including higher clinical efficacy, lower toxicity, and a broader spectrum of bactericidal activity (Shahid et al., 2009).

**FIGURE 1:**
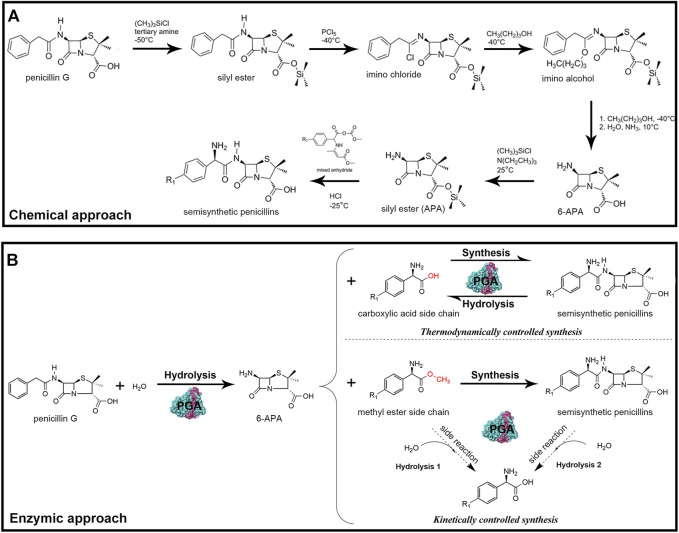
**(A)** Traditional chemical synthesis of β-lactam antibiotics. **(B)** Enzymatic synthesis of β-lactam antibiotics by PGA. PGA first deacylates penicillin G to produce key antibiotic nucleus 6-APA. Subsequently, PGA catalyzes the condensation of the nucleus and the acyl donor under thermodynamic or kinetic control, resulting in the generation of a series of semisynthetic penicillins.

Semisynthetic antibiotics are traditionally synthesized via a chemical process that involves hazardous chemicals and solvents, extreme temperatures, and regioselective protection and deprotection of functional groups (Figure 1A) (Verweij and De Vroom, 1993; Srirangan et al., 2013). In contrast, biosynthesis of antibiotics by penicillin G acylase (E.C.3.5.1.11; PGA) has great application potential for mild reaction conditions, high selectivity, and environmentally friendly character (Rajendhran and Gunasekaran, 2004). Since 1995, immobilized PGA has been used as an industrial biocatalyst for large-scale hydrolysis of penicillin G to produce key antibiotic nucleus 6-aminopenicillanic acid (6-APA) (Sudhakaran et al., 1992; Wenda et al., 2011). This enzyme has also been used to deacylate cephalosporin G, a compound derived from the ring-enlargement reaction of penicillin G, to produce another important nucleus labelled 7-amino-3-deacetoxy cephalosporanic acid (7-ADCA) (Verweij and De Vroom, 1993). Subsequently, through the PGA-catalyzed condensation of the corresponding nucleus and acyl donor, a series of semisynthetic penicillins and cephalosporins are obtained (Figure 1B) (Parmar et al., 2000; Wu et al., 2010).

**FIGURE 2:**
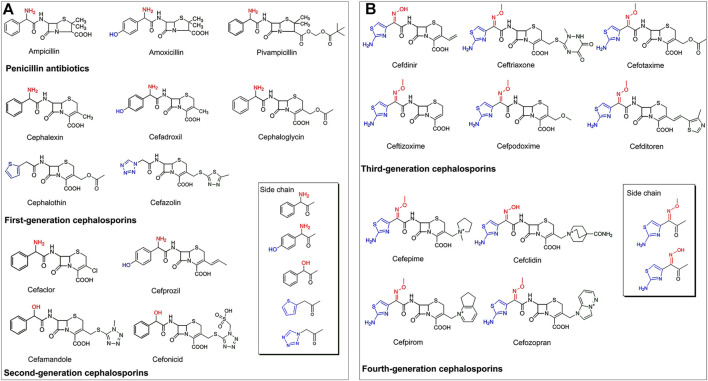
**(A)** The structure characteristics of PGA-catalyzed semisynthetic penicillins, first- and second-generation cephalosporins. **(B)** The structure characteristics of third- and fourth-generation cephalosporins.

Presently, the PGA-catalyzed biosynthesis of β-lactam antibiotics is mostly restricted to penicillin antibiotics, as well as first- and second-generation cephalosporins (Figure 2A). The side chains of these PGA-recognized antibiotics have the following two characteristics: (1) α-amino or α-hydroxy substituted phenyl acetic acid groups (e.g., ampicillin, amoxicillin, pivampicillin, cephalexin, cefadroxil, cephaloglycin, cefaclor, cefprozil, cefamandole, and cefonicid), and (2) thiophenyl or tetrazolyl acetic acid groups (e.g., cephalothin and cefazolin) (Srirangan et al., 2013; Marešová et al., 2014).

Based on the reaction types, the biosynthesis procedure is further divided into thermodynamic control or kinetic control (Cobos-Puc et al., 2020). In the first case, a non-activated acyl donor (free carboxylic acid) is used, and the synthesis is mainly governed by the reaction equilibrium constant, whereas the enzyme only accelerates the reaction rate by lowering the activation energy, which does not affect the product yield (Becka et al., 2014). Although this approach has been successfully applied in the synthesis of some antibiotics, its efficiency is extremely low. This is because the non-ionized active form of the acyl donor that is needed to shift the equilibrium toward synthesis is usually generated under conditions of high organic cosolvent concentration and low pH, which are hardly compatible with the activity and stability of PGA (Chandel et al., 2008). In the second case (kinetic control), the synthesis is mainly governed by the intrinsic properties of the enzyme, in which an activated acyl donor (ester or amide of carboxylic acid) is used (Dorr and Fuerst, 2018). Compared with the thermodynamically controlled counterpart, the kinetically controlled synthesis has a much higher yield and productivity, as the reaction is not restricted by equilibrium conversion, thus considered as the primary mean of antibiotics biosynthesis (Kurochkina and Nys, 2002). Despite the mentioned advantages, two side reactions occur alongside the main synthesis reaction of the desired antibiotic product, namely, primary hydrolysis of the activated acyl donor and secondary hydrolysis of the formed product (Figure 1B) (Volpato et al., 2010; Grulich et al., 2013). As a result, the maximum yields achieved are transient and depend on the balance between the synthetase, esterase, and amidase activities of PGA (Youshko et al., 2002).

In this review, we discuss the strategies adopted to improve the efficiency of PGA-catalyzed β-lactam antibiotic synthesis in recent 20 years. These strategies mostly rely on the enhancement of enzymatic property by the mining of enzymes and protein engineering, as well as on the optimization of the reaction system via solvent engineering, *in situ* product removal, and one-pot reaction cascade. These advances set the stage for realizing the large-scale application of antibiotic biosynthesis on an industrial level.

## Improving the Enzymatic Property

The intrinsic properties of the biocatalyst have a major impact on the efficiency of enzymatic synthesis of β-lactam antibiotics (Wang et al., 2007). In the case of kinetically controlled biosynthesis, the most desirable properties of PGA are low hydrolytic activity for acyl donors and high synthetic activity for antibiotic products. The former is quantified based on the synthesis/hydrolysis (S/H) ratio, which equals the ratio of the initial rate of the product synthesis to the initial rate of free acid formation. The S/H ratio determines the interaction preference of the acyl-enzyme intermediate, nucleophilically attacked by the β-lactam nucleus or by water. The higher this ratio, the more favorable the reaction with the β-lactam nucleus, and the higher the yield of the antibiotic product (Youshko et al., 2002; Ribeiro et al., 2005). Meanwhile, the latter is quantified based on the α value, which equals the ratio of enzyme specificity constants for antibiotic hydrolysis to that of acyl donor. The α value determines the selectivity between product hydrolysis and activated acyl donor hydrolysis. The lower the value of α, the greater the accumulation of the antibiotic product (Alkema et al., 2002a).

### Mining of Enzymes

**FIGURE 3:**
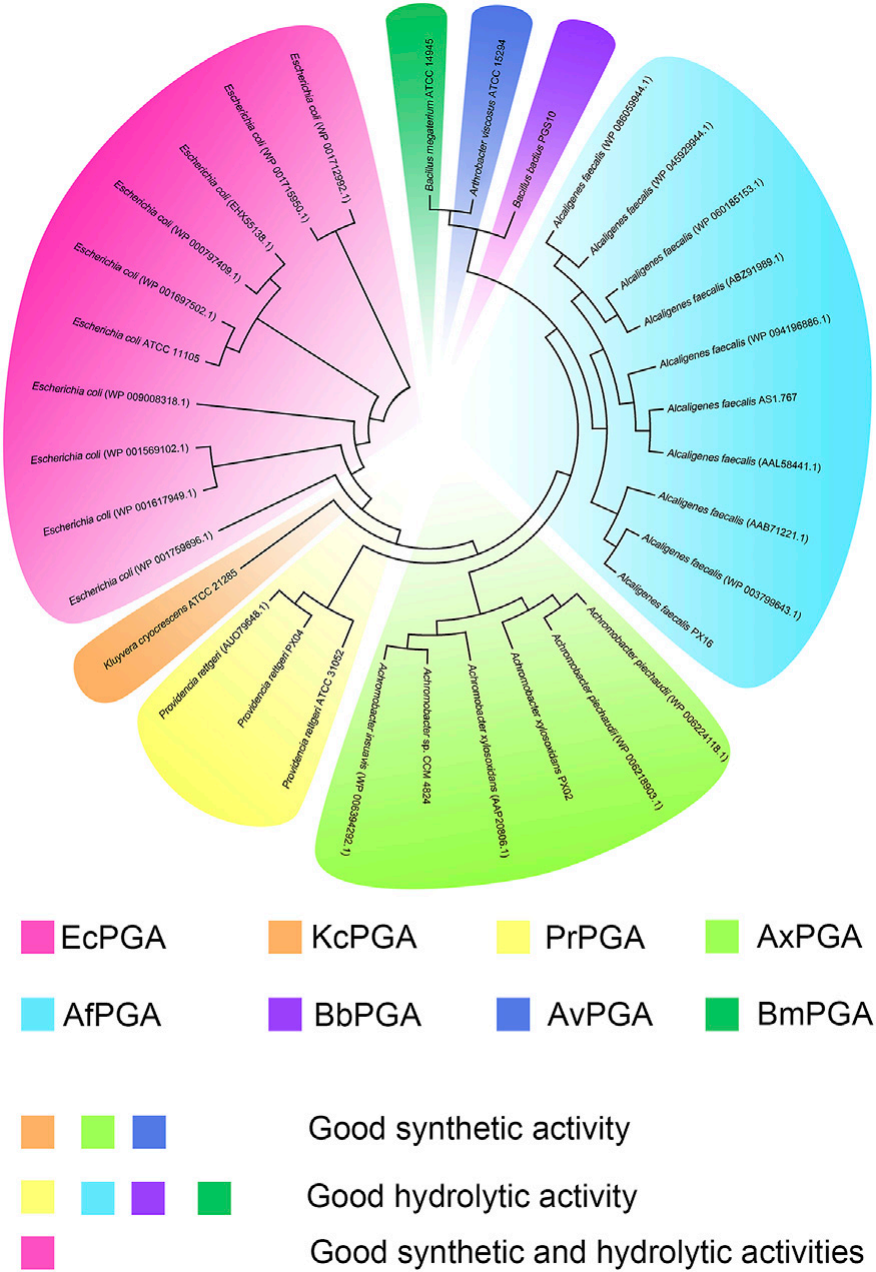
The phylogenetic tree of industrially relevant PGAs reported in the literature and their strain origins.

**FIGURE 4:**
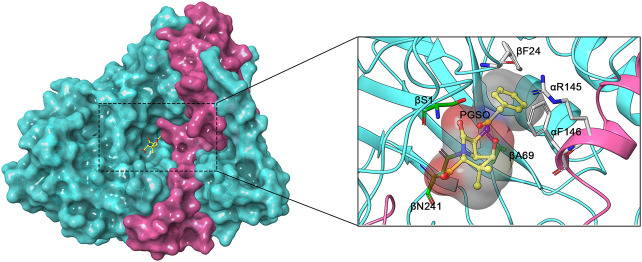
Crystal structure of EcPGA with ligand penicillin G sulfoxide (PGSO) (yellow) (PDB ID: 1GM9). The α and β subunits are coloured fuchsia and cyan, respectively. The active sites βS1, βA69, and βN241 are coloured green and good mutation sites αR145, αF146, and βF24 are coloured light grey.

In 1950, Sakaguchi and Murao reported a new enzyme isolated from *Penicillium chrysogenum* for the first time. This enzyme could hydrolyze penicillin G to produce 6-APA, and thus, it is named penicillin acylase (Sakaguchi, 1950). Thereafter, an increasing number of penicillin acylases have been identified in more than 40 microbial sources from bacteria, actinomycetes, and fungi (Tishkov et al., 2010). Based on their substrate specificity, penicillin acylases are further divided into three classes: PGA, penicillin V acylases (PVA), and ampicillin acylases (APCA) (Arroyo et al., 2003). Specifically, PGA preferentially hydrolyzes benzylpenicillin or penicillin G, PVA preferentially hydrolyzes phenoxymethyl penicillin or penicillin V, and APCA preferentially hydrolyzes d-α-aminobenzylpenicillin or ampicillin (Li et al., 2020). Among them, PGAs are more commonly used in industries and laboratories than PVAs and APCAs, and they are mostly produced by bacteria. Usually, the mature PGA is a heterodimer that consists of α and β subunits (Figure 4) (Pan et al., 2018b). The PGAs associated with antibiotic production include the periplasmic enzymes from Gram-negative bacteria, such as *Escherichia coli* (EcPGA), *Kluyvera cryocrescens* (KcPGA), *Providencia rettgeri* (PrPGA), *Achromobacter xylosoxidans* (AxPGA), *Alcaligenes faecalis* (AfPGA), and the extracellular enzymes from Gram-positive bacteria, such as *Bacillus megaterium* (BmPGA), *Arthrobacter viscosus* (AvPGA) (Figure 3) (Grulich et al., 2013; Srirangan et al., 2013). Intriguingly, one PGA extracted from Gram-positive bacteria *Bacillus badius* (BbPGA) is an intracellular enzyme (Karthikeyan et al., 2011).

Due to the wide variation in the intrinsic properties of different PGAs, their synthetic activities, especially the S/H ratios, are significantly different (Table 1). EcPGA is the most industrially relevant PGA, and it has been successfully applied in the large-scale production of β-lactam nucleus 6-APA and 7-ADCA (Wenda et al., 2011; Srirangan et al., 2013). In addition, EcPGA shows good efficiency in the enzymatic synthesis of several β-lactam antibiotics under kinetic control. In a study conducted by Alkema et al., EcPGA is reported to display high synthetic activities for ampicillin, amoxicillin, cephalexin, and cefadroxil due to good S/H ratios (1.4–5.2) (Alkema et al., 2002a). Cheng et al. also compared the efficiencies of four different PGAs used to catalyze cephalexin synthesis, and their results indicate that EcPGA (S/H = 13 ± 2) and KcPGA (S/H = 17 ± 3) are more suitable for antibiotic synthesis than AfPGA (S/H = 2.5 ± 0.7) and PrPGA (S/H = 3.3 ± 0.3) (Cheng et al., 2006). Later, in 2004, another penicillin G acylase, AxPGA, was discovered. This enzyme is characterized by excellent thermal stability and a half-life (t_1/2_) of 55 min at 55 °C. Comparatively, the half-lives of EcPGA and AfPGA under the same conditions are 5 and 15 min, respectively (Skrob et al., 2003). Moreover, in the synthesis of ampicillin and amoxicillin, AxPGA presents significantly higher S/H ratios than EcPGA (Becka et al., 2014). In our previous studies, we evaluated the activities of three different PGAs in terms of ampicillin and amoxicillin synthesis. The obtained results show that the catalytic activity of AxPGA, whose S/H ratio is 1.42–1.5, is more prominent than the activities of PrPGA and AfPGA, whose S/H ratios is 0.45–0.84 (Pan et al., 2018a; Pan et al., 2020). Although AfPGA has a unique disulfide bridge in the β-subunit, resulting in relatively high thermal stability (t_1/2,55°C_ = 15 min) (Verhaert et al., 1997), the overall catalytic efficiency of this enzyme, as well as of PrPGA, is less than those of other reported PGAs detected in Gram-negative bacteria.

**TABLE 1:** Synthesis performances of the industrially relevant PGAs reported in the literature.

Origin: Strain	Characteristics	Products	Nucleus/Acyl Donor (mM)	S/H	α	References
*E. coli*	Industrial biocatalyst for large-scale production of 6-APA and 7-ADCA Relatively high synthetic activity in several β-lactam antibiotics	Ampicillin	15/30	1.4	16	Alkema et al. (2002a)
		Amoxicillin	15/30	1.7	22.2	Alkema et al. (2002a)
		Cephalexin	15/30	4.9	63.3	Alkema et al. (2002a)
		Cefadroxil	15/30	4.6	55.6	Alkema et al. (2002a)
		Cephalexin	133/267	13	—	Cheng et al. (2006)
*K. cryocrescens*	High synthetic activity	Cephalexin	133/267	17	—	Cheng et al. (2006)
*P. rettgeri*	Relatively low synthetic activity	Cephalexin	133/267	3.3	—	Cheng et al. (2006)
		Amoxicillin	150/50	0.45	7.6	Pan et al. (2018a)
		Cefadroxil	150/50	0.56	38.3	Pan et al. (2018a)
*A. xylosoxidans*	High thermal stability (t_1/2,55 °C_ = 55min) High synthetic activity in several β-lactam antibiotics	Ampicillin	15/25	3.8	36.1	Skrob et al. (2003)
		Amoxicillin	15/25	3.2	87.1	Skrob et al. (2003)
		Cephalexin	15/25	3.7	17.4	Skrob et al. (2003)
		Cefadroxil	15/25	2.5	49.8	Skrob et al. (2003)
		Ampicillin	150/50	1.5	17.6	Pan et al. (2020)
		Amoxicillin	150/50	1.42	13.3	Pan et al. (2020)
*A. faecalis*	Unique disulfide bridge in the β-subunit Relatively high thermal stability (t_1/2,55°C_ = 15 min) Relatively low synthetic activity	Ampicillin	300/100	0.49	36.6	Verhaert et al. (1997)
		Cephalexin	133/267	2.5	—	Cheng et al. (2006)
		Ampicillin	150/50	0.84	—	Pan et al. (2020)
		Amoxicillin	150/50	0.73	—	Pan et al. (2020)
*B. megaterium*	Low synthetic activity Mainly produce 6-APA and 7-ADCA	Cephalexin	133/267	2.3	—	Yang et al. (2001)
*A. Viscosus*	High synthetic activity	Cephalosporin C	10/50	13.0	—	Terreni et al. (2007)

BmPGA, a PGA that is extracellularly expressed at high levels in *Bacillus subtilis* WB600, is currently used in the hydrolysis of penicillin G and cephalosporin G to produce nucleus 6-APA and 7-ADCA (Illanes et al., 1994; Yang et al., 2001). Besides, BbPGA is also proven to be capable of efficiently converting penicillin G to 6-APA (Karthikeyan et al., 2011). By contrast, AvPGA has a higher S/H ratio than EcPGA (13 vs. 9.6) in the synthesis of cephalosporin C, indicating a good enzyme source with high synthetic activity (Terreni et al., 2007).

In general, the eight PGAs discussed above are the main species used in the biocatalytic process of antibiotics. However, due to differences in properties, some PGAs (e.g., PrPGA, AfPGA, BbPGA, and BmPGA) prefer the hydrolytic activity toward antibiotics, while some PGAs (e.g., KcPGA, AxPGA, and AvPGA) are considered to be more appropriate in the synthesis of the antibiotic product. Notably, EcPGA shows good performance in the hydrolysis of penicillin G, as well as in the synthesis of semisynthetic antibiotics. Although progress has been made in the mining of PGAs, the number of PGA with superior synthetic activity is still scarce. Additionally, the yield of antibiotic synthesis, catalyzed by wild-type PGA, does not meet the requirements of industrial applications. Therefore, it is very urgent to explore novel PGAs with higher synthetic activities.

### Protein Engineering

Protein engineering is an effective and feasible strategy used to improve the intrinsic properties of PGAs, thereby enhancing the efficiency of enzymatic antibiotic synthesis. As is well known, in the case of EcPGA, its active sites are composed of amino acid residues βS1, βA69, and βN241 (Duggleby et al., 1996; Arroyo et al., 2003). Based on the mutational assessment, some residues close to the binding sites, such as αR145, αF146, and βF24, have strong interactions with the substrate and are thus considered good mutant targets (Figure 4) (Alkema et al., 2002b; Jager et al., 2008). According to the induced-fit model of enzymes, the structure of EcPGA switches from closed to open conformation upon binding to large substrates (e.g., penicillin G), which is mainly driven by the moving away of αR145, αF146 from the binding site. Specifically, αF146 can assist in binding the thiazolidine ring of the substrate via van der Waals interaction, whereas αR145 is bonded with the carboxylate oxygen atom of the ligand via two or three water molecules (Alkema et al., 2002b; Alkema et al., 2004). The βF24 residue also plays an important role in substrate binding since hydrophobic interactions exist between this aromatic residue and the phenyl ring of the substrate (Alkema et al., 2002a). Therefore, most studies regarding site-directed mutagenesis of PGAs are based on αR145, αF146, and βF24 (Table 2).

**TABLE 2:** Effect of site-directed mutations on the synthesis performances of PGAs.

Origin: Strain	PGA mutants	Mutation Results	Products	Nucleus/Acyl Donor (mM)	S/H	α	Yield (%)	References
*E. coli*	βF24A	↑2- to 3-fold increased S/H ratios	Ampicillin	15/30	2.9	37.5	—	Alkema et al. (2002a)
			Amoxicillin	15/30	3.1	14.6	—	Alkema et al. (2002a)
			Cephalexin	15/30	15.8	43.5	—	Alkema et al. (2002a)
			Cefadroxil	15/30	15	70.8	—	Alkema et al. (2002a)
*E. coli*	αR145G	↑1.4- fold increased S/H ratio	Cefazolin	480/400	1.34	1.11	92	Wang et al. (2021)
*E. coli*	αR146A, αR146L	↑2.2- to 3-fold increased S/H ratios	Ampicillin	15/30	3.1	—	—	Alkema et al. (2002a)
			Ampicillin	15/30	4.2	—	—	Alkema et al. (2002a)
*E. coli*	αF146Y/βF24A	↑1.3- to 1.7-fold increased yields	Cephalexin	150/50	—	—	99	Cecchini et al. (2012)
			Cefaclor	150/50	—	—	99	Cecchini et al. (2012)
			Cefprozil	150/50	—	—	99	Cecchini et al. (2012)
*E. coli*	αM142F/βF24A/βS67A	↑16-fold increased S/H ratio	Cephradine	480/400	21.73	0.28	>99	He et al. (2018)
		↓22-fold decreased α value						
		↑1.2-fold increased yield						
*P. rettgeri*	βF24G	↑3.8- to 5.4-fold increased S/H ratios	Amoxicillin	150/50	2.41	0.028	95	Pan et al. (2018a)
		↓270- to 890-fold decreased α values	Cefadroxil	150/50	2.13	0.043	95.4	Pan et al. (2018a)
		↑2.2- to 5.5-fold increased yields						
*A. xylosoxidans*	βF24A	↑2.3-fold increased S/H ratios	Ampicillin	150/50	3.42	0.11	96.1	Pan et al. (2020)
		↓140- to 160-fold decreased α values	Amoxicillin	150/50	3.35	0.09	93.2	Pan et al. (2020)
		↑1.9-fold increased yields						
*A. xylosoxidans*	αR141A/αF142I/βF24G	↑4.3-fold increased S/H ratio	Cefamandole	65/50	4.3	0.16	85	Li et al. (2021)
		↓110-fold decreased α value						
		↑2.1-fold increased yield						
*A. faecalis*	βF24G	↑8.6-fold increased S/H ratio	Ampicillin	300/100	4.2	0.39	95	Deng et al. (2015)
		↓93-fold decreased α value						
		↑4.1-fold increased yield						
*B. megaterium*	βV24F+αY144R	↑1.9-fold increased yield	Cephalexin	133/267	6.8	—	59.0	Wang et al. (2007)

So far, site-directed mutagenesis studies have been conducted on four PGAs from Gram-negative bacteria (EcPGA, AfPGA, PrPGA, and AxPGA), in which the αR145, αF146, and βF24 amino acid residues are completely conserved. According to Alkema et al., mutations in αF146 and βF24 residues of EcPGA increase the S/H ratio by 2- to 3-fold (Alkema et al., 2002a; Alkema et al., 2002b). Among them, βF24A appears to be the most efficient mutant with respect to the synthesis of several antibiotics. In the meantime, a significant decrease in α values is observed when esters are used as acyl donors, indicating that not only enzymatic properties but also substrate structural features play an important role in determining the S/H ratio of PGA. In a subsequent study, a more effective βF24A/αF146Y mutant is achieved by doubly mutating the αF146 and βF24 residues, leading to higher conversions in the synthesis of cephalexin (99% versus 76%), cefaclor (99% versus 65%), and cefprozil (99% versus 60%) compared with the wild-type enzyme (Cecchini et al., 2012). Further analysis shows that the acyl binding site on EcPGA structure consists of a hydrophobic pocket that has high affinity toward phenyl acetic acid groups. However, when α146 Phe is substituted with Tyr, higher affinity toward Cα-substituted phenyl acetic acid derivatives can be obtained due to van der Waals interactions between hydroxyl group of Tyr and the Cα-substituent. Meanwhile, the βF24A mutation has a conformation wherein the Cα-substituted phenyl acetic acid derivatives are tightly bound. All of these explain the reasons for the improved catalytic activity of βF24A/αF146Y mutant (Alkema et al., 2004).

In addition, site-saturating mutagenesis studies are performed on AfPGA (βF24, αR146, and αF147), PrPGA (βF24, αR143, and αF144), and AxPGA (βF24, αR141, and αF142). The results show that in the case of ampicillin synthesis, the βF24G mutant of AfPGA has an increased S/H ratio (4.2); however, the undesirable hydrolysis side reactions still occur due to the not sufficiently low value of α (0.39) (Deng et al., 2015). Unlike AfPGA, the βF24G mutant of the PrPGA enzyme has extremely low α values for amoxicillin (0.028) and cefadroxil (0.043) synthesis, resulting in a near complete absence of the hydrolysis product. Unfortunately, the S/H ratios of the βF24G mutant (2.13–2.41) are not outstanding (Pan et al., 2018a). In contrast, the obviously increased conversions (from 48.6%–51.2% to 93.2%–96.1%) with almost no product hydrolysis catalyzed by βF24A mutant of AxPGA are mainly attributed to the simultaneously attained higher S/H ratios (3.35–3.42) and lower α values (0.09–0.11), making this mutant possess striking properties. Molecular docking analysis shows that the active site pocket of βF24A mutant is enlarged remarkably, contributing to the decreased hydrolytic activity of the mutant toward the product (Pan et al., 2020). Our recent study also suggests that a triple mutant αR141A/αF142I/βF24G from AxPGA shows better performance in cefamandole production with a yield of 85% since its α value decreases dramatically from 17.8 (wild-type) to 0.16 (mutant) (Li et al., 2021).

Unlike the PGAs isolated from Gram-negative bacteria, the important residues in BmPGA are βV24, αY144, and αF145, respectively. Although the S/H ratio of the βV24F/αY144R mutant of BmPGA is almost 3-fold that of the wild-type PGA (6.8 vs. 2.3), the yield of the mutant-catalyzed reaction (59%) is still much lower than those achieved with other PGA mutants (Wang et al., 2007). This may be attributed to the intrinsic properties of wild-type BmPGA, which strongly favors the antibiotic hydrolysis procedure rather than synthesis.

Lately, the computational protein design program PRODA (PROtein Design Algorithmic package), developed by the Zhu group, has been used to explore PGA mutants with high synthetic activity (He et al., 2018). First, by placing cephradine in the enzyme active site with a near-attack conformation, the simulated nucleophilic attack of the acyl-enzyme complex by 7-ADCA is constructed. After calculating the binding and folding energies of the 2289 mutants generated by singly, doubly, or triply mutating 11 residues (αM142, αF146, βF24, βY31, βT32, βV56, βF57, βS67, βF71, βW154, and βI177) in PGA, the most efficient triple αM142F/βF24A/βS67A mutant is identified efficiently. The best mutant exhibits excellent performance in terms of cephalexin synthesis, with S/H ratios up to 21.7 and an α value of only 0.28. Subsequently, a similar method is used to obtain another single mutant αR145G for cefazolin synthesis from an initial library containing 770 sequences by the computational enzyme design program PRODA (Wang et al., 2021). As a result, the S/H ratio is increased by approximately 40% compared with the wild-type, and up to 92% yield is achieved with a 1.8:1 molar ratio of acyl donor/nucleus. In general, considering the efficacy of computational design strategy, it will be an important future direction for high-throughput screening of PGA mutants in different antibiotics synthesis.

## Strategies to Improve the Reaction System

### Solvent Engineering

Non-aqueous systems have been extensively used in kinetically controlled antibiotic biosynthesis, which has many advantages, including the enhancement of substrate and product solubility and the improvement of the microenvironment for enzyme catalysis (Klibanov, 2001; Itoh, 2017; Pätzold et al., 2019). As we know, three reactions usually exist in antibiotic synthesis: product synthesis, primary hydrolysis of the acyl donor, and secondary hydrolysis of the product, and their yields are strongly dependent on the enzyme properties. Given this, organic solvents such as methanol, ethylene glycol, glycerol, ethyl acetate, acetonitrile, and *tert*-pentanol have been used as cosolvents to address these limitations (Table 3). The main purpose of these organic solvents is to restrict the hydrolysis side reactions and improve the synthesis yield by lowering water activity (Chen et al., 2008; Pan et al., 2008; Liu et al., 2017). Among them, the ethylene glycol-water system is regarded as particularly appropriate in the kinetically controlled enzymatic synthesis of antibiotics. Studies on the ampicillin synthesis in ethylene glycol-water show that the incorporation of the ethylene glycol increases the S/H ratio and yield of the reaction, compared with the purely aqueous system, due to the reduced hydrolytic activity of the PGA (Illanes and Fajardo, 2001; Wei and Yang, 2003). Moreover, Deaguero et al. further revealed that during the process of ampicillin synthesis in ethylene glycol-water, the PGA catalyzes the transesterification of d-phenylglycine methyl ester (D-PGME) and ethylene glycol to form d-phenylglycine hydroxyethyl ester (D-PGHEE), a compound that also can function as an acyl donor and participate in the reaction (Deaguero and Bommarius, 2014). Compared with ethylene glycol, glycerol appears to be produced biologically and is completely non-toxic, cost-effective, and readily degradable, which is considered another suitable solvent. In the patent application for the enzymatic synthesis of cefaclor submitted by the DSM company, glycerol is listed as the preferred solvent, with a synthesis yield >95% (Moody et al., 2011). By combining the glycerol-water system and protein engineering, Deng et al. achieved 93.5% conversion at the D-PGME/6-APA ratio of 1.05:1, which is on par with chemical synthesis (Deng et al., 2016b).

**TABLE 3:** Effect of solvents on the kinetically controlled antibiotics synthesis by PGA.

Origin: Strain	Reaction Mediums	Products	Yield (%)	References
*E. coli*	45% (v/v) Ethylene glycol	Ampicillin	55	Illanes and Fajardo (2001)
*E. coli*	30% (v/v) Ethylene glycol	Ampicillin	45	Deaguero and Bommarius (2014)
*B. megaterium*	40% (v/v) Ethylene glycol	Ampicillin	52	Wei and Yang (2003)
*E. coli*	50% (v/v) Ethylene glycol	Cephalexin	72.3	Aguirre et al. (2002)
*E. coli*	60% (v/v) Ethylene glycol	Cephalexin	99	Illanes et al. (2004)
*E. coli*	20% (v/v) Ethylene glycol	Cefadroclor	76.5	Liu et al. (2017)
*E. coli*	Ethyl acetate	Ampicillin	92.9	Pan et al. (2008)
*E. coli*	10% (v/v) Methanol	Amoxicillin	—	Chow et al. (2007)
*E. coli*	*tert*-Pentanol	Amoxicillin	88	Chen et al. (2008)
*A. faecalis*	92% (v/v) Acetonitrile	Ampicillin	86	van Langen et al. (2003)
*A. faecalis*	15% (v/v) Glycerol	Ampicillin	93.5	Deng et al. (2016b)
Multispecies	10–30% (v/v) Glycerol	Cefaclor	>95	Moody et al. (2011)
*E. coli*	71% (v/v) Ionic liquids BMI·NTf_2_	Amoxicillin	—	Pereira et al. (2012)
*E. coli*	70% (v/v) Deep eutectic solvent choline chloride:glycol (1:2)	Cefaclor	91	Wu et al. (2019)

In recent years, ionic liquids composed of organic cations and inorganic or organic anions (at or near room temperature) have emerged as a class of new green solvents. Compared with conventional organic reagents, ionic liquids are less volatile, more soluble, and thermally stable. Considering their favorable properties, ionic liquids have been widely used as solvents in enzyme-catalyzed reactions (Itoh, 2017). In a study conducted on the effect of ionic liquids on the PGA-catalyzed synthesis of amoxicillin, Pereira et al. showed that these solvents effectively suppress the hydrolysis side reactions. Compared with a purely aqueous system, the S/H ratio and yield of the reaction, carried out in 71% (v/v) 1-butyl-3-methylimidazolium triflimide (BMI NTf_2_) ionic liquid, are 3.5 times and 36% greater (Pereira et al., 2012). Moreover, as ionic liquids analogue, deep eutectic solvent (DES) is considered cheaper, highly tunable, and more biodegradable (Pätzold et al., 2019). Based on the DES with choline chloride:glycol-buffer (7:3, v/v), Wu et al. catalyzed the synthesis of cefaclor with a higher S/H ratio (1.24 vs. 1.8) and yield (75% vs. 91%), showing the great potential of DES as an organic solvent alternative (Wu et al., 2019).

In summary, solvent engineering effectively improves the performance of PGAs in the synthesis of β-lactam antibiotics. To select the suitable solvent, the synthesis yield of the reaction, as well as the tolerance, stability, environmental friendliness, and cost-effectiveness of the enzyme, must be considered.

### *In situ* Product Removal

While kinetically controlled synthesis using an active acyl donor effectively improves the yield of antibiotic production, problems such as product inhibition, product secondary hydrolysis, and difficulty in product-substrate separation are encountered. *In situ* product removal (ISPR) eliminates these problems and greatly enhances the synthesis yield by continuously removing the products formed from the reaction system. The ISPR technique contains a number of approaches, such as complexation, aqueous two-phase system separation, and reaction at high substrate concentration, all of which have been successfully used in the enzymatic synthesis of antibiotics (Hülsewede et al., 2019).

Aqueous two-phase systems (ATPS) are prepared by mixing two hydrophilic polymers or one polymer with salt in water (Chao and Shum, 2020). The mutual exclusion between polymers ensures that polymer molecules of the same type always aggregate and that other types of polymers are excluded, giving rise to two phases at equilibrium. By adjusting the partition coefficient of the products and substrates in the two phases, the newly formed product molecules can be continuously removed from the reaction phase to effectively minimize their enzymatic hydrolysis and, thus, improve the antibiotic synthesis yield (Chao and Shum, 2020).

Previously, the aqueous two-phase systems, like PEG400-(NH_4_)_2_SO_4_, PEG600-(NH_4_)_2_SO_4_, and PEG400-MgSO_4_ (PEG: polyethylene glycol), have been applied to the synthesis of cephalexin (Table 4) (Hernandez-Justiz et al., 1998; Wei et al., 2002; Cao et al., 2004). The results show that all systems increase the product yield and reduce hydrolysis to different degrees. In particular, when the system is 100% PEG600-3M (NH_4_)_2_SO_4_, the final yield achieved is as high as 90%, which is much larger than the 55% yield obtained in a pure aqueous system (Hernandez-Justiz et al., 1998). In another study conducted by Terreni et al., they demonstrated that an 88% yield of cephalosporin intermediate may be achieved by the enzymatic synthesis in 80% PEG600-4M (NH_4_)_2_SO_4_ aqueous two-phase system (Terreni et al., 2005). To make the ATPS more reusable, novel polymers, such as the thermosensitive poly(NIPA-co-BA) copolymer (P_NB_) and pH-sensitive poly (AA-coDMAEMA-co-BMA) copolymer (P_ADB_), that can be precipitated and separated from the reaction system by regulating the temperature and pH, have been tested by Zhu group (Zhu and Cao, 2014). They achieved a final yield of 75.81% for cefprozil in ATPS consisting of (P_NB_/P_ADB_)-(NH_4_)_2_SO_4_, compared with only 56.02% yield in a pure aqueous system. By adjusting the solution temperature to 33 °C and the pH to neutral, the two polymers are successfully recovered (>97% recovery) for reuse. Clearly, the ATPS technique has great potential as a reusable system for the biosynthesis of antibiotics.

**TABLE 4:** Application of ISPR in the synthesis of β-lactam antibiotics by PGA.

Origin: Strain	ISPR Conditions	Products	Yield (%)	References
Aqueous two-phase systems				
*E. coli*	20% PEG 400–17.5% (NH_4_)_2_SO_4_	Cephalexin	53	Cao et al. (2004)
*E. coli*	20% PEG 400–15% MgSO_4_	Cephalexin	60	Wei et al. (2002)
*E. coli*	80% PEG 600–2.5M (NH_4_)_2_SO_4_	Cephalexin	78.2	Aguirre et al. (2010)
*E. coli*	100% PEG 600 - 3M (NH_4_)_2_SO_4_	Cephalexin	90	Hernandez-Justiz et al. (1998)
*E. coli*	80% PEG 600 - 4M (NH_4_)_2_SO_4_	Cephalosporin intermediate	88	Terreni et al. (2005)
*E. coli*	(P_NB_/P_ADB_) - (NH_4_)_2_SO_4_	Cefprozil	75.81	Zhu and Cao (2014)
Complexation				
*B. megaterium*	1-naphthol	Cefaclor	85	Yang and Wei (2003)
*E. coli*	1,5-dihydroxy-naphthalene	Cephalexin	74	Schroën et al. (2002)
*K. cryocrescens*	ZnSO_4_	Amoxicillin	76.5	Zhang et al. (2010)

Complexation is also an effective ISPR method that precipitates the antibiotic product formed from the solution (Table 4). Knowing that cephalosporin antibiotics such as cephalexin and cefaclor form insoluble complexes with naphthol and its derivatives so as to be removed from the reaction system and suppress product secondary hydrolysis (Yang et al., 2004). In previous antibiotic biosynthesis study, Yang et al. used *1*-naphthol to achieve complexation and precipitation of cefaclor, which leads the yield to increase from 64% to 85% (Yang and Wei, 2003). Similarly, Schroën et al. showed that a 74% yield of cephalexin is obtained when *1*,*5*-dihydroxynaphthalene is added to the reaction system (Schroën et al., 2002). Additionally, metal ions can form complexes with antibiotic products as well. A study on the complexation of amoxicillin with various metal ions has been investigated, and it seems that Zn^2+^ is appropriate for forming a low-solubility complex with amoxicillin. Taking this approach, Zhang et al. increased the synthesis yield of amoxicillin to 76.5% by complexation with Zn^2+^ (Zhang et al., 2010). Unfortunately, the Zn^2+^ and antibiotic complex does not precipitate, and thus, it cannot be readily separated from the solution.

Compared with ATPS and complexation, reaction at high substrate concentration contributes to the product accumulation and even direct precipitation in a pure aqueous system, which simplifies product downstream recovery (Li et al., 2008). The accumulation of product is induced by the increased probability of nucleus attack on the acyl-enzyme complexes at higher substrate concentrations and by the reduced water attack probability, which ultimately increases the S/H ratio of PGA and inhibits product hydrolysis (Youshko et al., 2004; Bahamondes et al., 2012). As shown in Table 5, the yield of cephalexin increases from 60% to 90% as the proportion of acyl donor is increased from 300 to 500 mM at 100 mM nucleus concentration (Shaw et al., 2000; Schroën et al., 2001). Further increasing the concentration of acyl donor and nucleus to 200 and 600 mM results in a 99% yield of cephalexin (Illanes et al., 2007). Unfortunately, in many high substrate concentration cases, a large quantity of by-product (mainly free acid form of acyl donor) often precipitates alongside the antibiotic product, which causes the *in situ* removal of both product and by-product. Youshko et al. used EcPGA to catalyze ampicillin synthesis in a heterogeneous aqueous solution–precipitate system containing 450 mM 6-APA and 600 mM D-PGME. Although a high yield (93%) is achieved, the ampicillin product and the d-phenylglycine (D-PG) by-product are both precipitated, and thus, they cannot be readily separated (Youshko et al., 2000). Later, a more effective strategy is adopted, wherein 650 mM 6-APA and 900 mM d-phenylglycine amide (D-PGA) are used in a stable homogeneous supersaturated system that achieves 98% yield. Unfortunately, the ampicillin product and the D-PG by-product are still precipitated together in this system (Youshko et al., 2004). Similarly, Deng et al. reported that the D-PG is formed in excess and is poorly soluble in the AfPGA mutant-catalyzed ampicillin synthesis. As a result, the reaction solution always tends to coagulate (Deng et al., 2016b). Even worse, Youshko et al. showed that excess acyl donors can increase the solubility of products in the reaction system. When 100 mM 6-APA and 500 mM D-PGME are used, a large quantity of by-product precipitates at the end of the reaction while the products remain in solution. By further increasing the 6-APA concentration from 100 to 300 mM, large quantities of both product and by-product can be precipitated at the end of the reaction, resulting in a higher final yield (87%) (Youshko et al., 2000). Hence, to avoid the precipitation of by-products, the amount of by-products formed by PGA-catalyzed hydrolysis should be kept low so that more products can be precipitated from the reaction system. In our previous study, we successfully determined an AxPGA mutant βF24A, which achieves final ampicillin and amoxicillin yields of 99.1% and 98.7% at 600 mM nucleus and 660 mM acyl donors, respectively. Most importantly, almost no by-product precipitation is observed, and the product purity is above 99% (w/w). This is the first realization of antibiotic synthesis with *in situ* product removal that is free from by-product precipitation (Pan et al., 2020). Afterward, in the case of cephalexin synthesis conducted by Fan et al., they also obtained a 99.5% (w/w) precipitated product with 99.3% yield by using suspension aqueous solution system at 659 mM nucleus concentration and 738 mM acyl donor (Fan et al., 2021).

**TABLE 5:** Synthesis of β-lactam antibiotics by PGA using high substrate concentration.

Products	Nucleus (mM)	Acyl Donor (mM)	Yield (%)	Precipitation Form	References
Cephalexin	100	300	60	Product and by-product	Shaw et al. (2000)
Cephalexin	100	500	90	Product and by-product	Schroën et al. (2001)
Cephalexin	200	600	99	Product and by-product	Illanes et al. (2007)
Cephalexin	659	738	99.3	Almost product	Fan et al. (2021)
Amoxicillin	600	660	98.7	Almost product	Pan et al. (2020)
Amoxicillin	650	900	91	Product and by-product	Youshko et al. (2004)
Ampicillin	100	500	75	Product and by-product	Youshko et al. (2000)
Ampicillin	300	500	87	Product and by-product	Youshko et al. (2000)
Ampicillin	400	420	93.5	Product and by-product	Deng et al. (2016b)
Ampicillin	600	660	99.1	Almost product	Pan et al. (2020)
Ampicillin	650	900	98	Product and by-product	Youshko et al. (2004)
Ampicillin	600	900	91	Product and by-product	Youshko et al. (2001)
Ampicillin	450	600	93	Product and by-product	Youshko et al. (2000)

### One-Pot Reaction Cascade

The enzymatic synthesis of semisynthetic antibiotics is usually accomplished in two steps. In the first step, the antibiotic nucleus, such as 6-APA or 7-ADCA, are obtained via the hydrolysis of penicillin G and cephalosporin G, and in the second step, the obtained nucleus undergoes an amidation reaction with different acyl donors to form a series of antibiotic products (Cobos-Puc et al., 2020). However, the recovery of 6-APA is only about 85% in the actual production process (Parmar et al., 2000; Rodriguez-Herrera et al., 2019). This problem may be resolved by cascading the reaction steps in one reactor (termed one-pot reaction cascade), thereby eliminating the need for intermediate product separation and purification, which ultimately renders the reaction more environmentally friendly and efficient (van Oers et al., 2014). Compared with the two-step synthesis, the one-pot method gives higher yield within a much shorter period. Importantly, this method greatly simplified the post-treatment steps of 6-APA.

**FIGURE 5:**
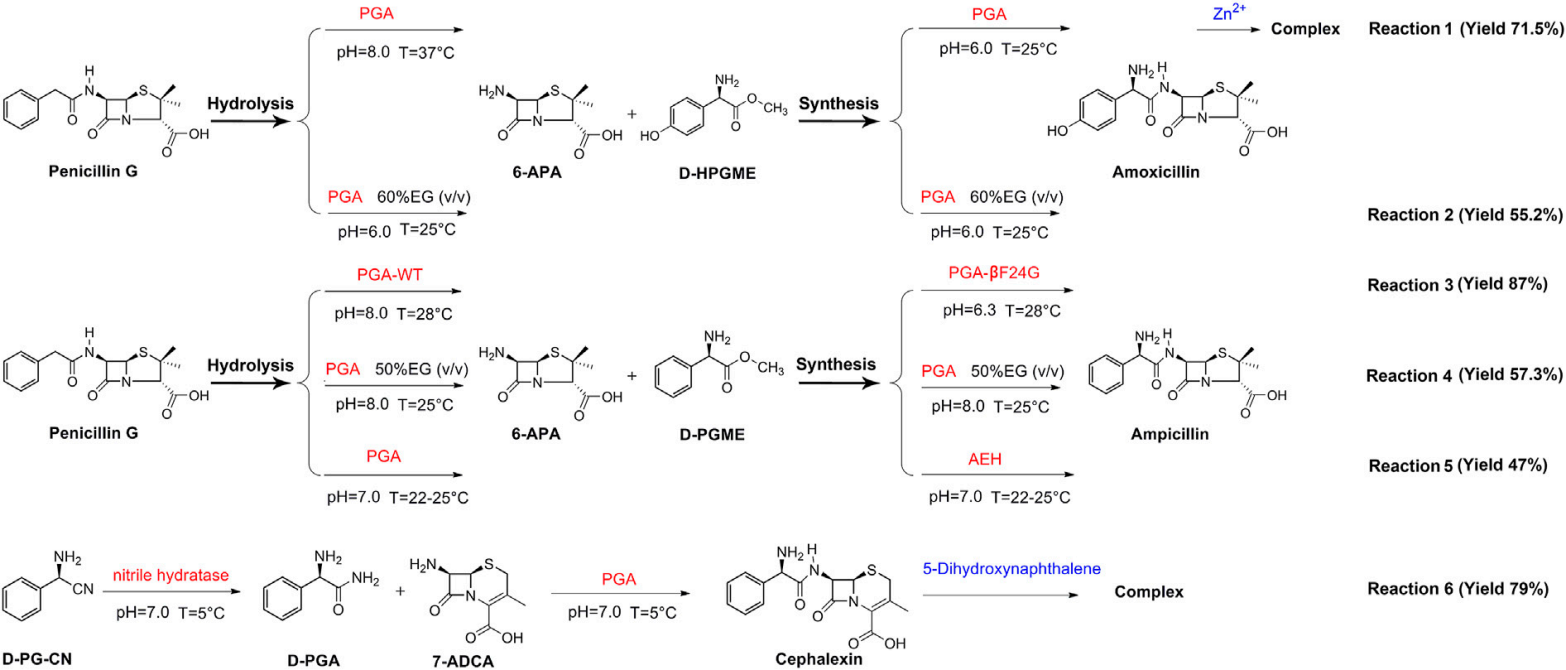
Application of one-pot reaction cascade in the synthesis of β-lactam antibiotics by PGA.

Presently, the one-pot reaction cascade has been successfully applied in the enzymatic synthesis of β-lactam antibiotics (Figure 5). In one such study (reaction 1), penicillin G is first hydrolyzed by PGA at 37°C under the alkaline condition to obtain 6-APA, then amoxicillin is synthesized at 25°C under acidic conditions. Zn^2+^ complexation is also applied in this study, resulting in a final yield of 71.5% (Zhang et al., 2010). Likewise, reactions 4 and 2 also make use of PGA with the addition of organic solvents to the reaction systems, eventually resulting in 57.3% and 55.2% yields, respectively (Du et al., 2009; Wu et al., 2010). It is noteworthy that the hydrolysis and synthesis of all three reactions are carried out only by PGA.

By contrast, reactions 3, 5, 6 are catalyzed using a combination of two enzymes as catalysts. Among them, both reactions 5 and 6 are performed at neutral pH (pH 7.0). In the case of reaction 5, penicillin G is hydrolyzed by PGA to obtain 6-APA, then 6-APA is reacted with D-PGME, using α-amino ester hydrolase (AEH) as a catalyst to form ampicillin at the final yield of 47% (Blum et al., 2010), whereas in reaction 6, nitrile hydratase is first used to hydrolyze d-phenylglycine nitrile (D-PG-CN) into D-PGA, then D-PGA is reacted with 7-ADCA under PGA catalysis to synthesize cephalexin. After complexation with 1,5-dihydroxynaphthalene, a final yield of 79% is obtained (Wegman et al., 2002). For reaction 3, the hydrolysis step is carried out using wild-type PGA under basic conditions, while the synthesis step is catalyzed by the PGA mutant βF24G under acidic conditions. The final yield of ampicillin is found to be 87% (Deng et al., 2016a).

The studies detailed above show that two conditions must be met in order to achieve efficient catalysis of one-pot enzymatic antibiotic synthesis. First, the enzymes used in hydrolysis and synthesis must have good catalytic performance in the relevant reactions. It means the enzymes with superior hydrolytic activity should be used in hydrolysis, while those with prominent synthetic activity should be used in synthesis. Although PGA can catalyze both hydrolysis and synthesis of antibiotics, the synthesis performance of wild-type PGA is always less efficient. Therefore, the use of PGA as a catalyst in both hydrolysis and synthesis reaction steps results in low yields (e.g., reactions 4 and 2). Considering that the synthesis performance of PGA mutants is better, the joint use of wild-type and mutant PGAs leads to a higher yield (e.g., Reaction 3). Second, the reaction conditions adopted in the hydrolysis and synthesis steps must be different. It is well known that high-temperature alkaline conditions facilitate hydrolysis, while low-temperature acidic conditions are more favorable for synthesis (Alekseev, 2010). When the two reaction steps are carried out under the same conditions, the production cost is low, but so is the final yield (e.g., reactions 2, 4, 5). Finally, the yield of the reaction can be improved by combining solvent engineering and complexation in a one-pot reaction system (e.g., reactions 1 and 6).

## Challenges and Future Prospects

The enzymatic synthesis of semisynthetic β-lactam antibiotics is a novel technique that can potentially replace traditional chemical synthesis. This technique produces little pollution and is relatively simple and cost-effective. The industrial production of 6-APA and 7-ADCA antibiotic nuclei has been successfully achieved using enzymatic hydrolysis. However, the industrial production of semisynthetic β-lactam antibiotics is still partly chemical, as enzymatic synthesis has not yet completely replaced the chemical methods. There are still many limiting factors for the PGAs’ industrial application, including (1) lack of excellent enzyme sources for different antibiotic products; (2) poor stability and lack of reusability of free enzyme; (3) an uneconomical acyl donor/nucleus ratio (excess acyl donor); (4) separation of the enzyme from the product.

Over the past 20 years, protein engineering has been widely used in the field of biocatalysis by improving the catalytic properties of enzymes (Wilding et al., 2019). Given this, for bottleneck 1, we consider that the computational design strategy is an extremely efficient approach to determine the mutants with improved synthetic activity (Wang et al., 2021). Of course, the complicated maturation process remains a technological hurdle for the heterologous expression of recombinant mutants (Gabor and Janssen, 2004). Thus, the combination of PGA expression strategies is essential and readers are referred to Srirangan’s excellent review of PGA expression (Srirangan et al., 2013). With respect to bottleneck 2, the immobilization of enzyme is still the most commonly used technique to improve the stability and reusability of the enzyme. The recent progress in the development of immobilized PGA for chemical and industrial applications has been detailedly reviewed by Li et al. (Li et al., 2020). In terms of the bottlenecks 3 and 4, on the one hand, the protein engineering and solvent engineering have been shown to effectively reduce the hydrolytic activity of PGA toward acyl donor. On the other hand, *in situ* product removal using high substrate concentration could enable product accumulation and direct precipitation, which simplifies the recovery of the enzyme from the product. Furthermore, the one-pot reaction cascade can significantly reduce the cost of industrial production of β-lactam antibiotics.

The emergence of resistance to antibiotics in bacteria is a worldwide health problem. It has necessitated the development of third- and fourth-generation cephalosporins that are characterized by higher antimicrobial activities compared with their first- and second-generation counterparts (Protic et al., 2016; Zhanel et al., 2019). However, to the best of our knowledge, the enzymatic synthesis of third- and fourth-generation cephalosporins by PGAs or other enzymes has not yet been reported. This may be attributed to the complex side chain structures of these antibiotics, most of which are characterized by α-hydroxyimino or α-methoxyimino substituted aminothiazole acetic acid derivatives that might not be effectively recognized by enzymes (Figure 2B). Therefore, more in-depth research is needed regarding the biosynthesis of more complex third- and fourth-generation cephalosporins.

Recently, artificial intelligence (AI) has shown great promise, especially in the fields of biomedicine and pharmacy (Yu et al., 2018). AI focuses on how computers learn from data and mimic human thought processes. It has successfully discovered a multitude of therapeutic targets as well as drug candidates (Topol, 2019). How to combine the biosynthesis process of β-lactam antibiotics with AI and to further enhance the catalytic efficiency of PGA. This may be a good research direction in the future.

## Conclusion

In this review, we discuss the strategies adopted to enhance the PGA-catalyzed biosynthesis of β-lactam antibiotics in the recent 20 years. These strategies are based on two main approaches: (1) improving enzymatic property and (2) optimizing the reaction system, whose advantages and disadvantages are compared. To improve the synthetic activity of PGA, protein engineering and mining of new enzymes are important approaches. In addition, the selection of a suitable reaction solvent is essential to improve the catalysis efficiency of PGA, as well as to decrease the environmental pollution. And *in situ* product removal not only improves the synthesis yield but also makes the separation of antibiotic products more convenient. For the one-pot reaction cascade, it can greatly reduce the cost of the whole reaction process. Considering that the enzymatic synthesis of β-lactam antibiotics is a highly complex process, it is essential to combine these strategies reasonably and effectively for practical and industrial application.
